# The Effects of Multi-Component Interventions on Cognition: A Systematic Review

**DOI:** 10.1093/geroni/igab046.2451

**Published:** 2021-12-17

**Authors:** Sangwoo Ahn, Joel Anderson

**Affiliations:** University of Tennessee at Knoxville, Knoxville, Tennessee, United States

## Abstract

Given the lack of a cure for Alzheimer’s disease (AD), the number of people with AD is expected to surge unless the onset is delayed. Although there have been efforts to examine the effects of single-domain neuroprotective interventions on cognition, no conclusive results have been found so far. Due to the multifactorial causes of AD, interventions combining multiple neuroprotective components may induce more beneficial effects. However, there are few comprehensive reviews evaluating the effects of multi-domain programs on cognition. Thus, the purpose of this systematic review was to evaluate the effects of currently available multi-component interventions on cognition such as global cognition, episodic memory, and/or executive function affected early in AD. The literature search was conducted using PubMed, CINAHL, Web of Science, Scopus, and PsycINFO up to September 2020. Of the 1,445 articles located, 17 met eligibility criteria (n = 10,056, mean age = 72.8 years). According to the Effective Public Health Practice Project Quality Assessment Tool for Quantitative Studies, 8 and 9 studies had strong and moderate overall quality, respectively. The effect sizes of each included study were calculated using Cohen’s d. Multi-component interventions comprising physical activity, cognitive exercise, cardioprotective nutrition, and/or cardiovascular health consultation/education exerted beneficial effects on cognition (very small to moderate effect sizes; Cohen’s d = 0.16 to 0.77). Clinically, health care providers are recommended to consider those elements to potentially stave off AD. There is a pressing need for researchers to identify optimally effective doses of neuroprotective multi-component interventions.

